# Implementation of plate reader-based indooxine and Nessler protocols for monitoring L-asparaginase serum activity in childhood acute lymphoblastic leukaemia

**DOI:** 10.1093/biomethods/bpae042

**Published:** 2024-06-13

**Authors:** Bozhidar Vergov, Yordan Sbirkov, Danail Minchev, Tatyana Todorova, Alexandra Baldzhieva, Hasan Burnusuzov, Мariya I Spasova, Victoria Sarafian

**Affiliations:** Department of Medical Biology, Medical University of Plovdiv, Plovdiv 4000, Bulgaria; Research Institute at Medical University of Plovdiv, Plovdiv 4000, Bulgaria; Department of Medical Biology, Medical University of Plovdiv, Plovdiv 4000, Bulgaria; Research Institute at Medical University of Plovdiv, Plovdiv 4000, Bulgaria; Department of Human Anatomy and Physiology, University of Plovdiv ‘Paisii Hilendarski’, 24 Tzar Asen St, Plovdiv 4000, Bulgaria; Department of Medical Biology, Medical University of Plovdiv, Plovdiv 4000, Bulgaria; Research Institute at Medical University of Plovdiv, Plovdiv 4000, Bulgaria; Research Institute at Medical University of Plovdiv, Plovdiv 4000, Bulgaria; Department of Medical Microbiology and Immunology ‘Prof. Dr Elissay Yanev’, Medical University of Plovdiv, Plovdiv 4000, Bulgaria; Research Institute at Medical University of Plovdiv, Plovdiv 4000, Bulgaria; Department of Pediatrics, Medical University of Plovdiv, Plovdiv 4000, Bulgaria; Pediatric Clinic, St George University Hospital, Plovdiv 4000, Bulgaria; Department of Pediatrics, Medical University of Plovdiv, Plovdiv 4000, Bulgaria; Department of Medical Biology, Medical University of Plovdiv, Plovdiv 4000, Bulgaria; Research Institute at Medical University of Plovdiv, Plovdiv 4000, Bulgaria

**Keywords:** L-asparaginase, therapeutic drug monitoring, acute lymphoblastic leukaemia, Nessler, indooxine, L-aspartic acid β-hydroxamate (AHA)

## Abstract

Monitoring the blood serum activity of L-asparaginase in children with acute lymphoblastic leukaemia (ALL) has been highly recommended to detect enzyme inactivation that can cause relapse and to avoid unwanted toxicity. Nevertheless, perhaps at least partially due to the lack of clinically approved commercially available kits or standardized and independently reproduced and validated in-house protocols, laboratory assay-based determination of the optimal doses of L-asparaginase is not carried out routinely. In this study, we adapted previously published protocols for two plate reader-based colorimetric methods, indooxine and Nessler, to measure asparaginase activity. Mock samples with dilutions of the enzyme for initial optimization steps, and patient samples were used as a proof of principle and to compare the two protocols. For the first time the indooxine and the Nessler methods are adapted for a plate reader and L-asparaginase serum activity levels are compared by both protocols. Passing–Bablok and Bland–Altman’s statistical analyses found very little difference, strong correlation (*r* = 0.852), and bias of only 6% between the data from the two methods when used for fresh patient samples. Furthermore, we demonstrate that the Nessler method could also be applied for frozen sera as the results, compared to fresh samples, showed little difference, strong correlation (*r* = 0.817), and small bias (9%). We successfully adapted and validated two methods for measuring L-asparaginase activity in cALL and provided the most detailed description to date on how to reproduce and implement them in other clinical laboratories.

## Introduction

Independent validation and successful repetition of protocols are notoriously poor across many scientific areas. Thus, around 70% of experiments in the field of biology are not reproducible, at least partially because of lack of sufficient methodological information or not entirely adequate description of the experimental design [1–3]. This, leads to considerable financial losses, estimated at nearly 30 billion dollars per year for preclinical research [4]. More importantly, however, lack of reproducibility or insufficient description of protocols may discourage the use of otherwise important diagnostic, prognostic, or drug monitoring methods in everyday clinical practice.

One example is the therapeutic monitoring of L-asparaginase activity in the context of childhood acute lymphoblastic leukaemia (cALL). Acute lymphoblastic leukaemia (ALL) is the commonest cancer type in children [5]. Even though the key components of standard chemotherapy have not changed over the past decades, improved therapeutic regimens nowadays provide above 90% 5-year survival rates for the age group 0–14 years and above 75% for adolescents (age 15–19) [5, 6]. An essential part of the therapeutic protocols is the enzyme L-asparaginase (ASNase), which hydrolyses asparagine (Asn) to aspartate (aspartic acid) and ammonium. Downregulation of asparagine synthetase in cALL lymphoblasts renders them dependent on extracellular import of Asn and vulnerable to ASNase [7].

Since the enzyme is derived from bacterial sources (mainly from *Escherichia coli* and *Erwinia chrysanthemi)*, it is a highly immunogenic protein and its repeated intravenous or intramuscular application can lead to the development of immune response [7, 8].

Several studies reported different incidences (up to 70%) of anti-ASNase antibodies formation in treated patients [8–10]. The immunoglobulins can mediate a hypersensitive reaction and compromise the therapy. They can bind to the enzyme, inactivate it or accelerate its clearance from the patient’s bloodstream without any clinical manifestations. Both events are summarized in the literature under the term ‘silent inactivation’. While allergic reactions vary in their severity and can be treated with antihistamines and corticosteroids, silent inactivation can dramatically affect the treatment without any hints to the clinicians [7, 8]. The high incidence antibody-mediated adverse events compel the use of therapeutic drug monitoring (TDM) and individualized therapy [11–15].

TDM assays involve mainly the measurements of: enzyme activity, antibody titre, and asparagine level. Anti-ASNase antibodies measurement is predominantly used for research purposes and it does not reflect the treatment efficiency. Asparagine detection in patient’s blood sample is complicated, and the ongoing asparagine depletion *ex vivo* makes this technique inaccurate. Therefore, measuring serum asparaginase activity level is recommended by an expert consensus as the method of choice, since it is reliable, reproducible and, most importantly, correlates best with the clinical effect of the therapy [15].

**Figure 1.**
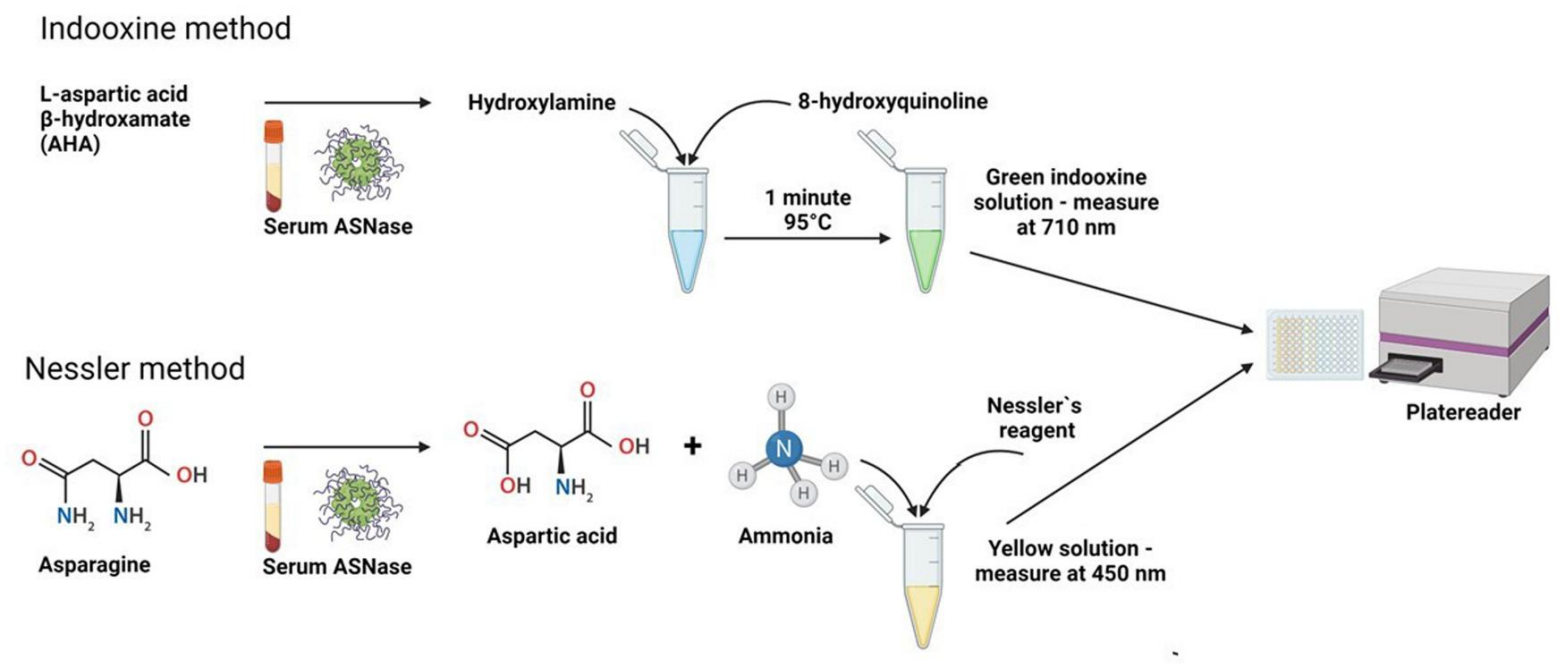
Schematic representation of the Nessler and indooxine methods for determination of serum L-asparaginase activity. Top: the indooxine method relies on the deamination of an artificial substrate for L-asparaginase—Laspratic acid β-hydroxamate, to hydroxylamine which can react with 8-hydroxyquinoline to produce the green indooxine chromogen. Bottom: the Nessler method measures ammonia released by L-asparagine that is added to patient samples by producing a yellow chromogen (amidoiodomercury). Figure was created in BioRender.com

Today, after the discontinuation of Medac’s L-asparaginase activity test (MAAT) kit, there is no commercially available IVD-CE-certified test that children’s hospitals in Europe could use. Concomitantly, at the time we started this work (end of 2021), we found a very limited number of published reports demonstrating optimized and validated laboratory protocols that are applied on a daily basis to inform clinicians of the therapeutic efficacy of this key drug in the BFM protocols. Based on our initial literature search, we decided to focus on two main plate reader-based methods (Fig. 1).

The first detects asparagine deamination by measurement of ammonia with Nessler’s reagent. We managed to find only two types of protocols—one developed by a group in the Czech Republic (2004) using ammonia for the standard curve [16], and one used in Denmark (2001, 2014, and 2017) [17–19] and Italy (2004) [20], relying on enzyme calibrated standard curve.

The second commonly used method for TDM of asparaginase utilizes an artificial substrate for the enzyme—L-aspratic acid β-hydroxamate (AHA), which is hydrolysed to aspartic acid and hydroxylamine, the latter being detected with the help of chromogens like indooxine. A pioneering study using this strategy was from Muenster, Germany published in 2002 [21].We further found a subsequent investigation by the same group in 2010 [22], a multicentre AIEOP-BFM ALL 2009 study, led again by the Muenster team, comparing the MAAT kit to the indooxine method (2018)[23]. Research carried out by a group in Brazil (2020) validating their results in the pioneering laboratory in Germany [24], and two reports from India—with the MAAT kit (2018)[25] and with indooxine (2020) [26] are the only publications, known to us, that are accessible in the scientific literature so far.

Thus, until recently, only a handful of published methods for measuring L-asparaginase activity were available for laboratories at children’s hospitals, mainly from clinics in Germany and Denmark. Therefore, given the lack of commercially available tests and widely used protocols for TDM of asparaginase, the aim of this study was to adapt and validate the indooxine and Nessler methods for the needs of the Children’s clinic at St George University Hospital in Plovdiv (Bulgaria). Here, we present a detailed description of both assays for ease of reproduction by other clinics. We also investigated how preservation of serum at −80°C may affect the data obtained by the two tests and discuss the advantages and disadvantages of both.

## Materials and methods

### Patient samples

Twelve venous blood samples from 10 children diagnosed with ALL were collected with informed consent signed by the parents/guardians. Ten of the samples were collected 2 weeks after infusion of pegylated L-asparaginase (Oncaspar^®^, Servier), one sample was collected 1 week after the infusion, and one sample was collected 2 days after infusion with Erwinase^®^ (Porton Biopharma) in a patient who had demonstrated an allergic reaction and had gone into relapse (patient LeuB1-R). All samples were collected in CAT (S-Monovette^®^ CAT/2.7 ml) monovettes and were immediately transported to the laboratory. Serum was obtained through centrifugation at 400*g* for 5 min. 200 µl of the serum was used for ASNase activity measurement and at least 200 µl was stored at −80°C for further analysis. Patient characteristics are summarized in Table 1.

### Statistical analysis

For the calculation of standard curves and interpolation of unknown asparaginase activity values of the tested samples, we implemented a simple linear regression model. The *R*^2^ value was used to assess the goodness of fit. These statistical analyses were carried out with GraphPad Prism (version 10.2) [23]. А Passing–Bablok regression was applied to compare the analytical methods [i]. The regression analysis was performed using the ‘mcreg’ function of the ‘mcr’ package in R version 4.3.2. The ‘MCResult.calcCUSUM’ function of the ‘mcr’ package was used to confirm the linearity of each according to Passing and Bablok [27]. A Bland–Altman statistical approach was applied to account for bias [28].

### Indooxine method (step-by-step protocol)

This method is based on the conversion of L-aspratic acid β-hydroxamate (AHA) by the enzyme into L-aspartic acid and hydroxylamine. The latter, together with 8-hydroxyquinoline produces a green indooxine dye. All reactions take place in 1.5 ml Eppendorf tubes and the final readings are carried out in standard 96-well plates. All required reagents and their used concentrations are:

1. A 10 mM AHA solution (cat# 0000132892, Sigma-Aldrich, USA) in deionized water, supplemented with 0.015M Tris buffer pH 7.4 (cat# 3818008, Sigma-Aldrich, USA), and 0.015% w/v bovine serum albumin (BSA) (cat# SLBW3872, Sigma-Aldrich, USA).
2. 24.5% w/v Trichloroacetic acid (TCA) (cat# STBK4799, Sigma-Aldrich, USA) (in water),
3. 2% 8-hydroxyquinoline (cat# 210520, Sigma-Aldrich, USA) solution in absolute ethanol.
4. 1M disodium bicarbonate (cat# BCCF6908, Sigma-Aldrich, USA) (in water)
5. For the standard curve—serial dilutions (in 0.05M Tris buffer) of L-asparaginase (sc-470689, Santa Cruz Biotechnology, Inc, USA or Oncaspar^®^, Servier, France for patient samples where available) with the following concentrations—0.1, 0.2, 0.4, 0.6, 0.8, and 1 IU/ml.

First, 20 µl of patient serum (or L-asparaginase solution for the standard curve) is added to 180 µl 10 mM solution of AHA, which is a substrate for the enzyme ASNase. After 30 min of incubation at 37°C, the reaction is stopped by adding 250 µl of TCA. Samples are centrifuged at 2000*g* for 5 min. Then 10 µl of the supernatant, which contains the converted by the enzyme hydroxylamine, is transferred to a new Eppendorf tube where it is diluted with 40 µl of deionized water. Following the addition of 200 µl from a solution containing three parts of disodium bicarbonate and 1 part of 2% 8-hydroxyquinoline (i.e. 150 µl 1M sodium carbonate and 50 µl of 2% 8-hydroxyquinoline per reaction), the tube is heated for 1 min at 95°C. The hydroxylamine and 8-hydroxyquinoline react to produce indooxine, which turns the solution green. Tubes are centrifuged at 2000*g* for 1 min, the green solution is transferred to a standard 96-well plate and the absorption is measured at 710 nm (Synergy/LX multi-mode reader, BioTek, USA).

For the preparation of the standard curve, L-asparaginase (Santa Cruz Biotechnology) was dissolved in 1 ml deionized water yielding 1 mg/ml or 1166 IU/ml, aliquoted and stored at −20°C. Aliquots should be used only once (freeze–thaw cycles are not recommended), and it is also important to note that a fresh standard curve should be prepared for each new sample from these aliquots. Alternatively, if available, several microlitres from a freshly prepared Oncaspar^®^ (750 IU/ml) could be obtained after it has been used for infusion and the standard curve could be prepared from it too (if stored refrigerated at 2°C to 8°C for no more than 48 hours as recommended by the manufacture). A 1:115.6 dilution is needed for the 1 IU/ml (e.g. 2 µl asparaginase and 131 µl deionized water containing final concentration of 0.05M Tris buffer) standard. When the remaining Eppendorf tubes with 0.8, 0.6, 0.4, 0.2, and 0.1 IU/ml are prepared, 20 µl from each tube is added to 180 µl 10 mM solution of AHA and the rest of the protocol is identical as for the preparation of patient samples. After all absorption readings have been obtained, the resulting values from the patient samples can be interpolated from the standard curve (as described above). The method is presented schematically in Fig. 1.

### Nessler method (step by step)

This method is based on a chemical reaction between ammonia, which is released after L-asparaginase hydrolyses and deaminates L-asparagine, and Nessler′s reagent (K_2_HgI_4_), which results in a yellow solution. All reactions take place in 1.5 ml Eppendorf tubes and the final readings are carried out in standard 96-well plates.

The reagents needed to be prepared in advance are:

1. 44 mM L-asparagine (cat# SLCJ1974, Sigma-Aldrich, USA) solution in deionized water.
2. 50 mM Tris buffer (cat# 3818008, Sigma-Aldrich, USA).
3. 24.5% w/v Trichloroacetic acid (TCA) (cat# STBK4799, Sigma-Aldrich, USA) (in water),
4. Nessler Reagent (Nessler’s Reagent A) (cat# HC15915611, Sigma-Aldrich, USA).
5. Sodium hydroxide (Nessler’s Reagent B) (cat# HC14942312, Sigma-Aldrich, USA).
6. For the standard curve—ammonium sulphate (cat#7783202, Sigma-Aldrich, USA) at different concentrations—0.2, 0.3, 0.4, 0.6, 0.8, and 1 mM, stored refrigerated at 2°C to 8°C.

First, two 1.5 ml Eppendorf tubes are prepared by adding 50 µl 44 mM L-asparagine solution, 450 µl deionized water and 500 µl 50 mM Tris buffer. To one of the tubes, which is going to be used to determine background readings of already released ammonium in the serum (not released after adding the L-asparagine substrate, but from the deamination of amino acids already present in the serum), 50 µl of TCA (24.5% w/v) is added in order to precipitate and inactivate the L-asparaginase. Then, 50 µl of serum is added to both tubes and they are incubated for 45 min. at 37°C. The reaction in the second tube is stopped after 45 min. by adding 50 µl TCA. The tubes are centrifuged at 2000*g* for 5 min. Then, 25 µl of the supernatant, containing released ammonia, from each tube are added to different wells in a 96-well plate (in duplicates or triplicates). Two hundred microlitres of deionized water are added to each well.

A fresh standard curve is prepared for each new sample as 100 µl of every ammonium sulphate solution is mixed with 100 µl 50 mM Tris buffer and 5 µl TCA (24.5% w/v) in 1.5 ml Eppendorf tubes. Then 25 µl from each dilution is added to different wells (in duplicates or triplicates) of a 96-well plate, pre-filled with 200 µl deionized water. For quantification of the released ammonia in the patient samples and in the standard curve wells, 25 µl of Nessler reagent (1:1 mixture of Nessler’s Reagents A and B) is added to each well (final volume of 250 µl per well) and mixed carefully by pipetting. Finally, the absorption is measured at 450 nm.

For the standard curve, first the amount of ammonia (in moles) in the standard dilutions should be plotted on the X-axis versus the respective absorption on the Y-axis. Note that 1 mole of ammonium sulphate has 2 moles of ammonia, but we have a 1:1 dilution with 100 µl of water so that e.g. 1 mM of ammonium sulphate contains 2 mmoles of ammonia per litre, but when diluted 1:1 we would have 1 mmole per litre or 1 µmole/ml. It is very important to take into account that we use 25 µl of these dilutions per well, which means that this is only 1/40th of the moles that are in a millilitre, that is for the 1 mM dilution that contains 1 µmole/ml, the final number of moles in a well from the 96-well plate is 1/40th of 1 µmole or 0.025 µmoles. Thus, for a standard curve made from 0.2, 0.3, 0.4, 0.6, 0.8, and 1 mM stock solutions, the equivalent number of moles that should be plotted on the X-axis is 0.005, 0.0075, 0.010, 0.015, 0.020, and 0.025* *µmoles, respectively.

The calculation of the standard curve and interpolation of enzyme activity is performed as follows:

$$\mathrm{ASNase}\;\mathrm{activity}\;=\frac{X\;µ\mathrm{moles}\;\mathrm{ammonia}\;\times \;1.1ml}{0.025ml\;\times \;45\;\min\;\times \;0.05ml}$$

where **X** is the ammonia concentration interpolated from the absorption of the sample and the standard curve described above; **1.1** is the volume of the enzymatic reaction that takes place in the Eppendorf tube (50* *µl serum, 50* *µl 44* *mM L-asparagine solution, 450* *µl deionized water and 500* *µl 50* *mM Tris buffer, 50* *µl TCA); **0.025** is the volume of the reaction in millilitres (25* *µl), which is used in the 96-well plate for the measurement; **45** is the incubation time in minutes; **0.05** is the volume of serum in millilitres (50* *µl) used in the reaction.

Thus, the resulting number is the enzymatic activity of L-asparaginase in units (i.e. 1* *µmole of ammonia released per 1 min at 37°C) per millilitre.

Of note, good laboratory practice would require regular validation of the two methods by using mock samples prepared from L-asparaginase (e.g. 0.1, 0.4, and 1 IU/ml), which can be analysed with the Nessler protocol. This way both the enzymatic activity of the aliquots stored at −20°C and the calibration of the ammonium sulphate standard curve can be double-checked.

## Results

We first sought to investigate whether at least one of the two methods for the detection of serum asparaginase activity, via detection of ammonia and formation of indooxine, can be adapted to a simple, quick, reproducible, and reliable plate reader-based routine monitoring of therapy in cALL patients at the Paediatrics Clinic in St George University Hospital in Plovdiv (Bulgaria). As a starting point, we considered a commonly used protocol for indooxine detection found in several previous publications of clinical relevance most of which could be traced back to the original study of Lanvers *et al*. from 2002 [21]. However, we could not find even one well-described Nessler reagent-based method in the literature to monitor L-asparaginase activity in patient serum, especially with regard to calculating the final enzymatic activity. Therefore, we resorted to a manual for the ‘enzymatic assay of asparaginase’, published by Sigma-Aldrich in 1997, based on the original work of Shifrin *et al*. from 1974 [29].

**Figure 2.**
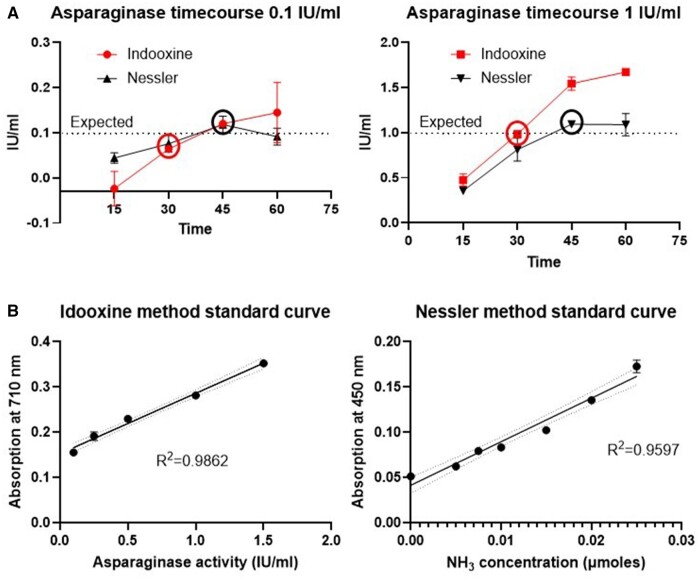
Timecourse of 0.1 and 1 IU/ml asparaginase and indooxine and Nessler standard curves. (**A**) L-asparaginase activity over time (15–60 min of incubation as indicated) for indooxine (red lines and square point symbol) and Nessler (black lines and triangle point symbol) methods with 0.1 IU/ml (left) and 1 IU/ml (right). The selected time of incubation for further analysis is indicated with circles at 30 min for the indooxine and 45 min for the Nessler methods. Graphs are representative of two ndependent experiments in duplicates. Error bars show standard error of means. (**B**) Standard curves for indooxine (left) and Nessler (right) methods built with simple linear regression. Dotted lines indicate the 95% confidence intervals.

In order to build reproducible and reliable standard operating procedures for both methods, the initial experiments aimed at validating the concentrations of substrates (both AHA and L-asparagine), as this is critical for the activity of asparaginase, and the incubation time of the enzyme. The clinically relevant range of serum asparaginase activity is between 0.1 IU/ml, which is based on the consensus expert recommendation [15], and 1 IU/ml, which are the peak pegylated (PEG)-asparaginase levels [14] (pegylation of the enzyme reduces immunogenicity and increases the half-life of asparaginase which makes forms like Oncaspar^®^ the first choice of therapy including in our small cohort of patients). Using these two values as lower and upper limits, we found that 30 min incubation for the indooxine method is a good compromise for both of these values. Of note, we detected that longer incubation times (45 min or 1 h) may result in overestimation of enzyme activity (especially with 1 IU/ml), which would be misleading for decision-making by clinicians (Fig. 2A). For the Nessler method, we confirmed that 45 min of incubation with the enzyme achieves results close to the expected for both 0.1 IU/ml and 1 IU/ml.

The standard curves for both methods showed acceptable linearity within the 0.1–1 IU/ml range (*R*^2^ = 0.9862 for indooxine and *R*^2^ = 0.9597 for Nessler) (Fig. 2B), which allowed us to proceed with the validation of these two methods with patient samples.

Using these optimized and validated protocols, we examined 12 samples from 10 children with B-cell cALL undergoing therapy according to the standard current BFM protocols. Following the recommendations for TDM of asparaginase, blood was collected 2 weeks after treatment with pegylated asparaginase (Oncaspar^®^) or 2 days after application of Erwinase^®^ (another form of the enzyme produced by the bacterium *Erwinia chrysanthemi*) for one patient who had previously demonstrated allergic reactions to the *E. coli* enzyme and had a relapse. The results and patient data are summarized in Table 1.

**Table 1.** Summary of patient data and L-asparaginase activity measured by the indooxine and Nessler methods in fresh and frozen samples.

Patient ID	Diagnosis	Age	Relapse	Indooxine (fresh) IU/ml	Nessler (fresh) IU/ml	Indooxine (frozen) IU/ml	Nessler (frozen) IU/ml
LeuB1	BCP-ALL	13	No	0.09	0.09	0.16	0.31
LeuB1-R	BCP-ALL	13	Yes	0	0	0	0
LeuB2	BCP-ALL	7	No	0.86	0.68	0.335	0.83
LeuB3-1	BCP-ALL	2	No	0.64	0.59	0.2	0.53
LeuB3-2	BCP-ALL	2	No	0.65	0.6	0.3	0.84
LeuB4	BCP-ALL	5	No	0.49	0.7	0.47	0.92
LeuB5	BCP-ALL	6	No	0.48	0.82	0.35	0.82
LeuB6	BCP-ALL	6	No	0.77	0.88	0.35	0.76
LeuB7	BCP-ALL	2	No	0.7	0.53	1.02	0.9
LeuB8	BCP-ALL	8	No	0.252	0.11	0.03	0.085
LeuB9	BCP-ALL	2	No	0.6	0.52	0.28	0.3
LeuB10	BCP-ALL	3	No	0.74	0.7	0.16	0.49

LeuB1-R: sample taken after relapse of the patient.

LeB3-1 and LeuB3-2 are two samples taken from the same patient at different time points during therapy (days 14 and 28); LeuB7 was taken 7 days after Asparaginase infusion.

Alt text: A table summarizing patient data like age, diagnosis, relapse, and results measured for asparaginase serum activity levels by the Indooxine and Nessler methods.

**Figure 3.**
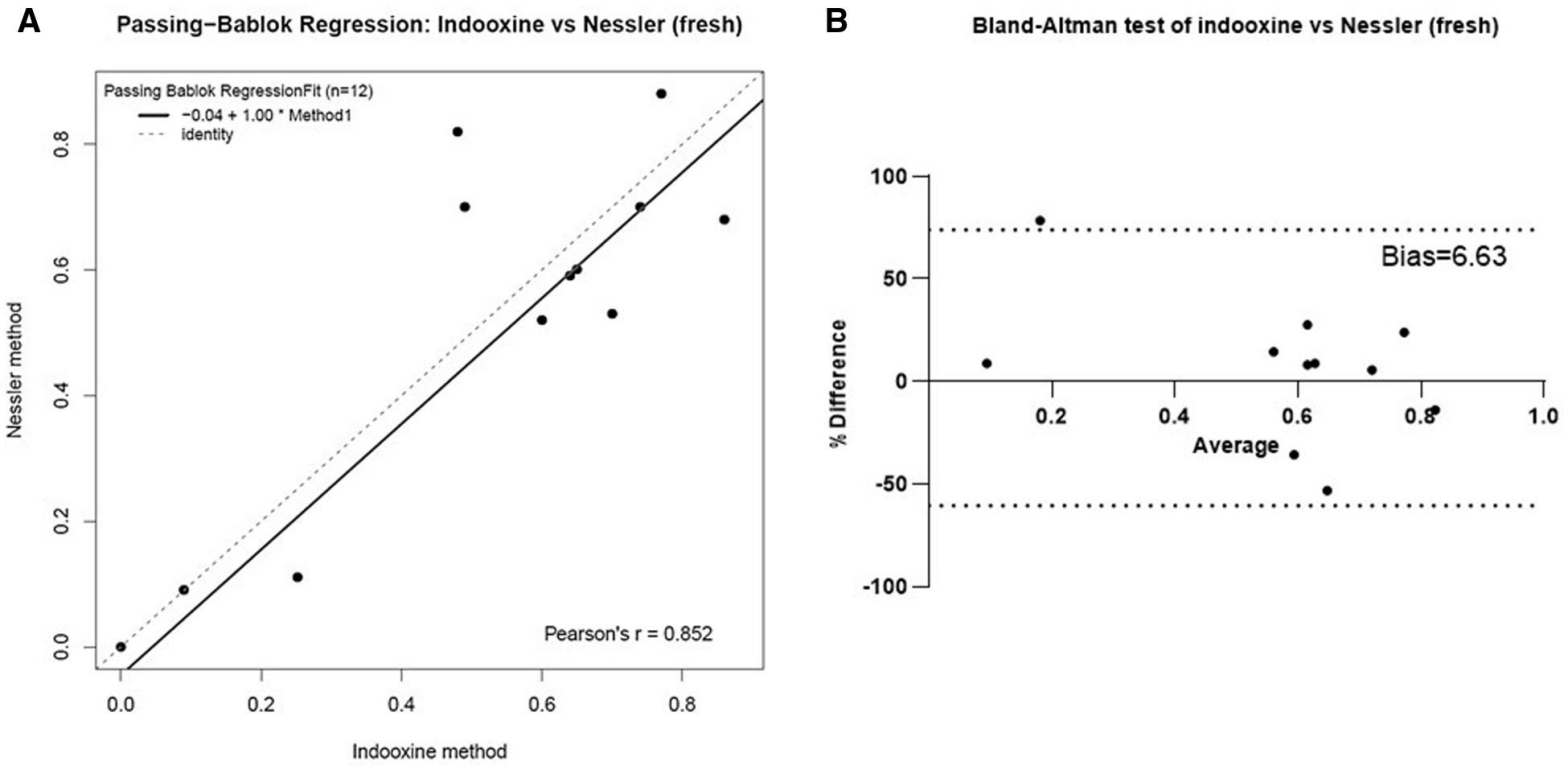
Comparison of the indooxine to the Nessler method. (**A**) Passing-Bablok regression scatter plot comparing between the results obtained from the indooxine and Nessler methods from fresh serum. (**B**) Bland–Altman test for similarity of the two methods. Dotted lines show the 95% limits of agreement.

In order to assess the similarity (or difference) between the data from the Nessler and indooxine methods and account for potential bias we carried out Passing–Bablok regression and Bland–Altman analyses. The Passing–Bablok test produced the following regression function: *y* = 1.00x − 0.04 (95% CI slope, −0.01, 1.80; 95% CI intercept, −0.51, 0.70). It revealed no significant constant difference. The methods were markedly identical with no statistically valid systematic difference in favour of any of them. Furthermore, the two methods appeared highly concordant, yielding results with significant correlation (Pearson’s *r* = 0.85) (Fig. 3A) The Bland–Altman analysis also demonstrated very little difference and only a small bias (6%) towards higher readings from the indooxine method in fresh samples compared to the results measured with the Nessler test (Fig. 3B).

**Figure 4.**
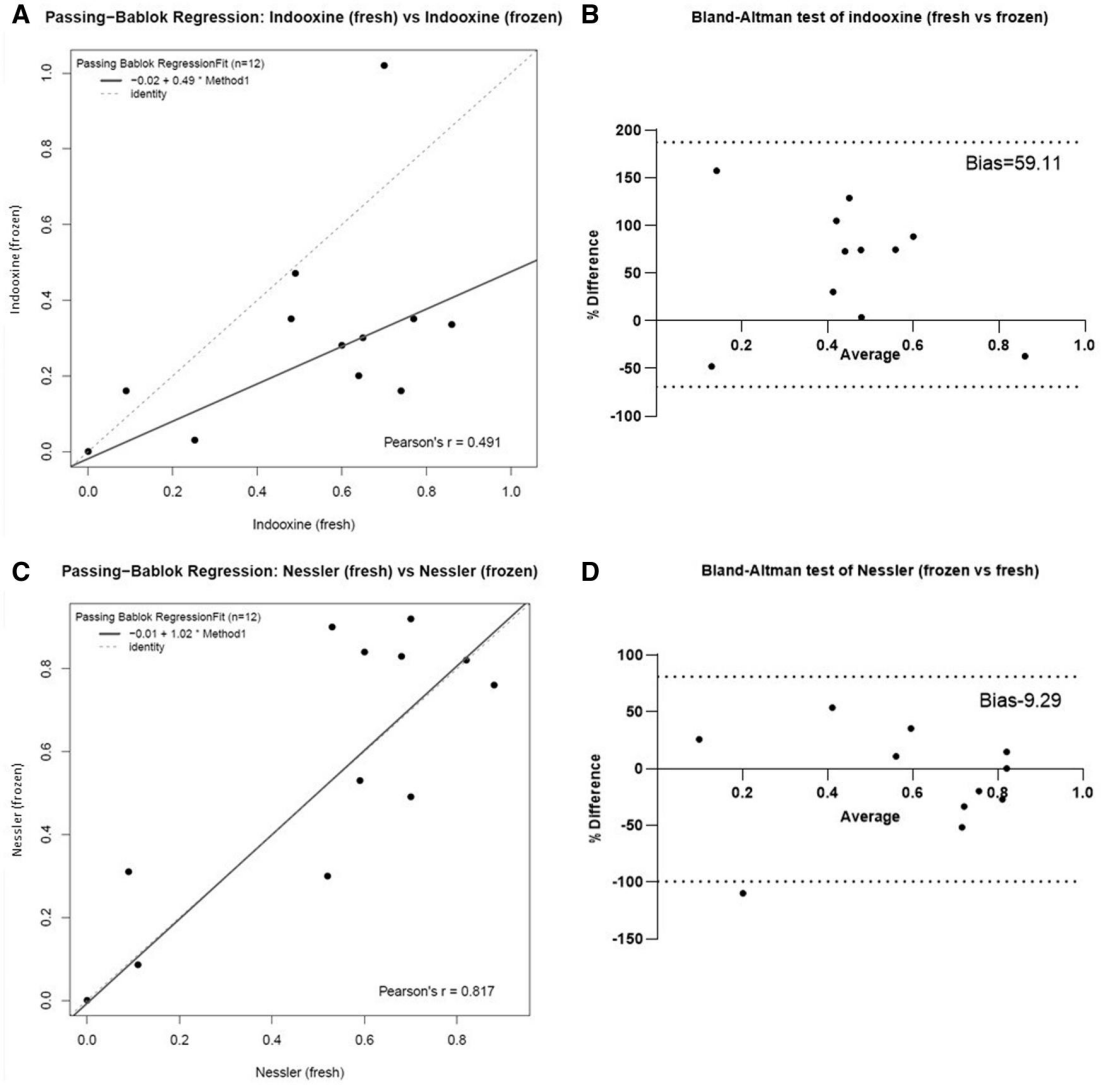
Comparison of the indooxine and Nessler methods in fresh and frozen sera. (**A**) and (**C**) Passing–Bablok regression scatter plots comparing fresh and frozen sera datapoints obtained by the indooxine and Nessler methods, respectively. (**B**) and (**D**) Bland–Altman tests for similarity of the methods using fresh or frozen samples. Dotted lines show the 95% limits of agreement.

Next, we attempted to investigate whether frozen samples could be re-analysed successfully. Again, aiming to obtain an objective assessment of the similarities and differences between the two datasets, we carried out Passing–Bablok and Bland–Altman tests (Fig. 4). The regression comparison between fresh versus frozen samples tested according to the indooxine protocol yielded the following function: *y* = 0.49x − 0.02 (95% CI slope, −1.06, 2.61; 95% CI intercept, −1.04, 0.99). The obtained confidence intervals of slope and intercept suggest that these parameters differ from one and zero only by random chance [30]. Additionally, the observed correlation between methods is relatively weak, with a Pearson’s *r* of only 0.49 (Fig. 4A). The Bland–Altman test also showed considerable differences with a bias of 59% towards lower readings obtained from frozen samples (Fig. 4B). In contrast, we managed to reproduce to a large degree the enzyme activity values measured from fresh serum with the Nessler method when samples were thawed. When the Passing–Bablok regression was used to compare fresh and frozen samples, the resulting regression function possessed a slope and intercept with no significant difference from one and zero, respectively: *y* = 1.02x − 0.01 (95% CI slope, −0.46, 1.40; 95% CI intercept, −0.89, 1.73). The between-method correlation analysis determined a strong agreement between measurement methods: Pearson’s *r* = 0.82 (Fig. 4C). Lastly, the Bland–Altman analysis also demonstrated a relatively little difference between the two datasets obtained from fresh and frozen sera (9.29%) (Fig. 4D).

## Discussion

Inefficient treatment or discontinuation of the therapy with asparaginase can lead to relapse, especially in high-risk patients, which makes the TDM of this enzyme of high importance [15, 31]. Nevertheless, the lack of commercially available and validated patient sample kits, as well as the insufficient description of methodology, make measuring serum asparaginase activity widely inaccessible by Children’s hospitals (at least in Europe) [32]. A unique survey in Spain from 2023 covering 90% of Children’s hospitals treating cALL demonstrated that more than 50% of clinics are not monitoring asparaginase activity due to lack of available laboratory testing in 80% of the cases [32]. Furthermore, 95% of the participants would prefer that the TDM be carried out in their own centres highlighting the significance of the need to implement ready-to-use, quick, cheap and robust in-house protocols.

Our work began at the end of 2021 when we expected a similar widespread lack of TDM of asparaginase serum levels across Europe since Medac had discontinued their IVD-certified kit. Our literature search at the time returned essentially only two centres measuring enzyme activity routinely—Germany and Denmark [17–19, 21, 22]. In Bulgaria, there were no available protocols for in-house testing so we here present the first description of validated indooxine and Nessler methods. Interestingly, one study from Poland was published after the start of this work using the MAAT kit (2022) [33], a group from Hungary also shared their experience with a commercial non-IVD-CE certified kit sold by Sigma-Aldrich (but discontinued) [34]. Two more reports came out earlier this year—a brief report from a centre in Madrid [35] and another study was published in March by a Croatian team (using the indooxine method based on a previously published study [36, 37]. This research demonstrates the significance of the topic and places our work within an ongoing solution to a widely spread and significant problem.

In this study, we summarize our experience with adapting and implementing two protocols for plate reader-based routine TDM of asparaginase serum activity in cALL. The indooxine and Nessler methods are both very affordable, quick (they take around 2 h to execute in parallel), require simple equipment available in the vast majority of clinical laboratories, and give reliable results. The data obtained by both tests show insignificant differences, both statistically and clinically (Fig. 3). We also observed a strong positive correlation between the results from the two methods (Pearson’s *r* = 0.85). The Bland–Altman analysis demonstrated very little bias of only 6% (i.e. difference of 6% compared to the X-axis, which represents zero difference between the two tests [38]) (Fig. 3). Of note, this difference between the tests is much less than the one Lanvers-Kaminsky *et al*. has described between indooxine and MAAT measurements (37%) [23].

Importantly, another validation of the methods described here is that our results fit very well within most published reports with regards to median values at day 14 after asparaginase infusion (∼0.6 IU/ml for 10 of our 12 patients excluding a sample taken at day 7 and one where Erwinase^®^ was used) 0.6 IU/ml—approximately 0.35 IU/ml [34], 0.4 IU/ml [35], and 0.6 IU/ml [37]. Lastly, we also tested if frozen samples can be analysed with the idea that sera from other clinics may be shipped for analysis. Interestingly, we found that in our hands the Nessler protocol worked more consistently and reliably in frozen samples, which is the first demonstration of the possibility to use this method in such a way in a clinical context.

There are several strengths of this study. The first one is that, compared to other similar reports, we provide the most detailed step-by-step description of how to carry out the measurement of serum L-asparaginase. The adaptation of the protocols to standard plate readers available in any clinical laboratory, the fact that the reagents are not expensive, the relative simplicity of execution, and the total time of only about 2 h for both tests, would make these protocols widely accessible and allow reproduction and implementation by other laboratories too. The second strength of this work is that for the first time, we provide the calculations for constructing a standard curve with ammonia sulphate for the Nessler method which greatly facilitates the interpolation of the serum asparaginase activity, especially with software with such built-in functions like GraphPad Prism. Moreover, this is in contrast to previously published reports where the standard curve is based on serial dilutions of the enzyme [17–20]. Using ammonia sulphate, however, avoids the need to purchase asparaginase, reduces the risk of overestimation of the activity due to old or improperly stored aliquots of the enzyme, and makes the assay more consistent overall.

Compared to our protocols, Konecna *et al*. did not show any calculations and their protocol was adapted for a spectrophotometer rather than for a plate reader [16]). Importantly, the other advantage of the Nessler protocol we provide is the option to re-measure frozen samples or to ship such samples for analysis in another centre, which is supported by the Bland–Altman analysis showing a 9% difference in the results from fresh and frozen sera.

Regarding the Nessler method, the biggest concern in literature has been with deamination of amino acids in serum, since this method will detect any amine groups, regardless of whether they have been released because of L-asparaginase during the 45-min incubation period or spontaneously. Literature suggests that this is certainly true if samples are kept at room temperature or even at 4°C for a few hours [39]. Nevertheless, freezing and thawing for 1 cycle has not been shown to increase, for example glutamate levels significantly, offering the opportunity to ship fresh-frozen samples for analysis or re-measure samples after being thawed once. Freeze–thawing for three cycles, however, has been demonstrated to alter the levels of most amino acids [39] so then the Nessler method would most likely give higher values for enzyme activity.

Our work has several limitations as well. The biggest one is the small number of patient samples due to the fact that there are not many cases of cALL per year in the country overall and in the region in particular (average of seven patients per year [40]). Another aspect to be considered is the fact that 1 patient sample was obtained on day 7 after asparaginase infusion and 1 sample was measured after treatment with Erwinase^®^. The disadvantage of this is that the mean serum activity values for both of these types of samples would be suspected to be rather different compared to those obtained on day 14, which can distort the statistics, especially in such a small dataset. However, the protocols we describe here will be very clinically useful if implemented for this type of sample too (even if enzyme pharmacokinetics and *in vitro* kinetics may be different for Erwinase^®^) and including these two data points may also be considered an advantage.

Despite the small number of paired samples, the Passing–Bablok and Bland–Altman tests complement each other and produce unbiased and robust results. Taking into account the concordance between the statistical tests, we believe that herein we provide sufficient evidence to demonstrate that the data obtained from the indooxone and Nessler protocols (in fresh sera) are reproducible and reliable and of clinical significance (we had only one patient with the initial activity of 0.1 IU/ml who relapsed, while none of the other patients has relapsed). There are also three aspects of our indooxine protocol that failed to reproduce previously published literature. The first one is the significant reduction of measured asparaginase activity in sera stored at −80°C, which is in contrast to what groups in Brazil and Croatia had found when sending their samples (frozen) for independent validation in Germany and the Netherlands, respectively [24, 37]. Nevertheless, analysing the data by Cecconello *et al*. (Table 3) [24], we could find a small, but significant (*P *=* *.0351) decrease in the enzymatic activity when measured after thawing (difference of means of 0.024 IU/ml), even if this difference was much smaller than what we recorded in our small dataset.

Since we found no evidence in the literature that may suggest altered substrate specificity of the enzyme after freeze–thaw cycles and obtained consistent results with the Nessler method in frozen samples, we believe that enzyme activity is not affected significantly during storage at −80°C. Factors that may influence the indooxine method, however, include changes in pH, which can alter the stability of hydroxylamine [41], salinity of the solution, as well as nitrite content [42]. Since we have not modified much the indooxine method from the protocols we found in the literature, examining the reasons for the lower activity we measured is beyond the scope of this study. Even if the lower activity we recorded with the indooxine method is due to technical issues, other groups may have similar experience to ours, so we would recommend the protocol only for fresh samples.

The second and third aspects of the indooxine protocol that we could not reproduce are perhaps not too relevant from a clinical and practical point of view. We did not find the test to be as sensitive as to detect activity of 0.02 IU/ml as previously described. The standard curve for activity below 0.1 IU/ml did not show good linearity (data points between 0.01 and 0.1—data not shown). However, lower values than the recommended consensus threshold of 0.1 IU/ml [15] would still indicate ineffective therapy and require a readjustment of the dose or a switch to Erwinase^®^. We observed the same lack of sensitivity below these values with the Nessler method as well, even though the previously published reports used more and less diluted serum for their spectroscopy measurements (e.g. 100 µl diluted in 400 µl of substrate-buffer solution [43, 44]). Thus, it is likely that more optimization steps should be carried out, related to the substrate concentration, time of the reaction and others, and perhaps a separate protocol would have to be developed in case laboratories would like to implement more sensitive Nessler and indooxine tests. However, we consider these disadvantages of our protocols to be more technical rather than clinical as several reports suggest that 0.1 IU/ml may not be sufficient to deplete asparagine levels, and suggest 0.2 IU/ml [45] or even that 0.4 IU/ml may be a more suitable threshold, especially for high-risk patients [46]. Lastly, one study comparing mass-spectrometry to the indooxine and Nessler methods found that the latter actually overestimates the enzyme activity [47], which was also suggested by Lanvers *et al*. in spite of the fact that they observed an excellent correlation and a small Bland–Altman test difference between the two methods [21]. This, however, was not performed with patient samples and was carried out with very high enzymatic activities of 1 to up to 14 IU/ml, which are an order of magnitude higher than the clinically relevant and obtainable values at day 14 after administration. In fact, at 1 and 2 IU/ml, the differences between the methods were not obvious [47].

## Conclusions

Here, we provide detailed step-by-step reproducible protocols for measurement of serum L-asparaginase for the TDM of patients with cALL. In spite of a small sample size, the two statistical tests we applied show very little difference in the data obtained with the indooxine and Nessler methods, proving the robustness and reliability of both. The required reagents, time and equipment make the protocols widely accessible and allow implementation by other laboratories too. Even though we found that the Nessler method can be used in frozen samples too and is perhaps technically easier than the indooxine protocol as other authors have also concluded [48], we believe that both methods give adequate and reliable information to clinicians. The application of both protocols could help prevent toxicity and silent inactivation and relapse in a number of Children’s hospitals, which are currently not performing routine TDM of L-asparaginase for their patients.

## Data Availability

The data underlying this article are available in the article. *Conflict of interest statement*. None declared.
